# Expression and clinical significance of colorectal cancer stem cell marker EpCAM^high^/CD44^+^ in colorectal cancer

**DOI:** 10.3892/ol.2014.1907

**Published:** 2014-02-21

**Authors:** DAN LIU, JINGHUA SUN, JINMING ZHU, HUAN ZHOU, XIAN ZHANG, YANG ZHANG

**Affiliations:** 1 Department of Oncology, The Second Affiliated Hospital of Dalian Medical University, Dalian, Liaoning 116027, P.R. China; 2 Department of Oncology, Affiliated Zhongshan Hospital of Dalian University, Dalian, Liaoning 116001, P.R. China

**Keywords:** colorectal cancer, double immunohistochemical staining, EpCAM^high^/CD44^+^, stem cells

## Abstract

Colorectal cancer stem cells are considered the source of recurrence, metastasis and drug resistance in colorectal tumors. Therefore, the identification and targeting of cancer stem cells facilitates the elimination of tumors. Although epithelial cell adhesion molecule-high (EpCAM^high^)/cluster of differentiation (CD)44^+^ cells are thought to act as a marker of colorectal cancer stem cells, the clinical significance of these cells in colorectal cancer remains unclear. The aim of the present study was to explore the prevalence and clinical significance of colorectal cancer stem cell marker EpCAM^high^/CD44^+^ in colorectal cancer. Double immunohistochemical staining was used to detect the expression of EpCAM/CD44 in 80 cases of colorectal cancer and their corresponding liver metastases. The expression of EpCAM/CD44 in colorectal cancer was analyzed, and the correlation of EpCAM^high^/CD44^+^ with the biological behavior of colorectal cancer was explored. In the 80 cases of colorectal cancer studied, the presence of EpCAM^high^/CD44^+^ cells had no correlation with gender, patient age or the magnitude of the tumor (P>0.05), but was significantly correlated with degree of differentiation, depth of invasion, clinical stage and metastatic status (P<0.05). In addition, EpCAM^high^/CD44^+^ cells were detected in the corresponding liver metastases. Thus, the results of this study indicate that EpCAM^high^/CD44^+^, a marker of colorectal cancer stem cells, is significantly correlated with the invasion and metastases of colorectal cancer.

## Introduction

Colorectal cancer is one of the most common types of cancer and the second greatest cause of cancer-associated mortality in the United States (1). Epidemiological investigation has revealed the highest incidence of colorectal cancer in North America and Australia, suggesting it is a disease associated with lifestyle (1). With changing diet in China, the incidence and mortality of colorectal cancer are increasing markedly. Although surgical resection, radiotherapy, chemotherapy and targeted therapies are available for the treatment of colorectal cancer, recurrence and metastasis (20–40% of patients present with liver metastases at diagnosis) remain a cause of poor prognosis.

It has been proposed that a small group of cells exists in tumors with properties of stem cells, including self-renewal, multilineage differentiation, multidrug resistance and tumorigenesis. These cells are considered cancer stem cells and the source of the recurrence and metastasis of colorectal cancer (2,3). Previous studies have revealed that epithelial cell adhesion molecule-high (EpCAM^high^)/cluster of differentiation (CD)44^+^ colorectal cancer cells have such stem cell-like properties, leading to tumorigenesis, invasion and metastasis of colorectal cancer (4–6). This suggests that EpCAM^high^/CD44^+^ may be used as a marker of colorectal cancer stem cells (4).

In order to further understand the clinical significance of EpCAM^high^/CD44^+^ colorectal cancer cells, the present study used double immunohistochemical staining to detect the presence of EpCAM^high^/CD44^+^ cells in 80 cases of colorectal cancer and 10 cases of corresponding liver metastases to explore the correlation between these cells and the biological behavior of colorectal cancer.

## Materials and methods

### Patients and tissue samples

Clinical data were collected from medical records at the First and Second Affiliated Hospitals of Dalian Medical University (Dalian, China). Formalin-fixed paraffin-embedded tumor samples from 80 cases of colorectal cancer and 10 cases of corresponding liver metastases obtained between 2003 and 2010 were used in this study. In total, 30 cases of normal intestinal mucosa were used as the controls. Paraneoplastic intestinal mucosa samples were used as controls. There were 38 male cases and 42 female cases. Patient age ranged between 19 and 83 years (mean age, 60 years), and 44 cases were >60 years old and 36 were ≤60 years old. Tumor diameter in 30 cases was >5 cm and <5 cm in 50 cases. Histologically, there were 10 highly differentiated, 51 moderately differentiated and 19 poorly differentiated cases. According to tumor, lymph node and metastasis (TNM) staging, 18 cases were stage T1 or T2, 28 cases were T3 and 34 cases were T4. Dukes’ staging described 16 cases of Dukes’ A, 16 cases of Dukes’ B, 22 cases of Dukes’ C and 26 cases of Dukes’ D. Metastases were present in 48 cases and absent in 32.

All histological diagnoses were independently confirmed by two experienced pathologists. No patient had received chemotherapy or radiotherapy prior to surgery. All tumors were classified using the 2010 criteria of the World Health Organization (WHO) (7,8). The study was approved by the Regional Ethics Committee of Dalian Medical University and performed in accordance with the Declaration of Helsinki. Patients provided written informed consent.

### Double immunohistochemical staining

Embedded specimens were sectioned at a thickness of 4 *μ*m. Double immunohistochemical staining was performed according to DouSP™ double staining kit (MaiXin Bio, Fuzhou, China) procedures. Following deparaffinization and dehydration, endogenous peroxidases were blocked briefly with 3% H_2_O_2_. Non-specific antibody binding was blocked with goat serum for 10 min at room temperature. Sections were sequentially incubated with primary antibody [mouse anti-human EpCAM monoclonal antibody (MaiXin Bio)] overnight at 4°C, followed by the secondary antibody [goat anti-mouse/rabbit polyclonal IgG (MaiXin Bio)] for 15 min at room temperature. Sections were incubated with alkaline phosphatase for 15 min at room temperature. EpCAM was detected with 5-bromo-4-chloro-3-indolyl phosphate, *p*-toluidine salt (BCIP)/nitro blue tetrazolium (NBT) at room temperature. Positively stained cells exhibited a blue-black precipitate in the cytoplasm. Sections were subsequently incubated with double staining enhancement solution for 10 min at room temperature, then blocked with goat serum for 10 min, also at room temperature. Sections were then sequentially incubated with primary antibody [rabbit anti-human CD44 polyclonal antibody (Proteintech Group, Inc., Chicago, IL, USA)] overnight at 4°C and secondary antibody [goat anti-mouse/rabbit IgG (MaiXin Bio)] for 15 min at room temperature. CD44 was detected with aminoethylcarbazole (AEC) at room temperature. Positively stained cells exhibited red precipitate in the cytomembrane. In order to prove the reliability of double staining of EpCAM^high^/CD44^+^, immunohistochemical staining of EpCAM^high^/CD44^−^, EpCAM^low^/CD44^+^ and EpCAM^low^/CD44^−^, respectively were used as controls.

### Assessment of double immunohistochemical staining

**Figure 1:**
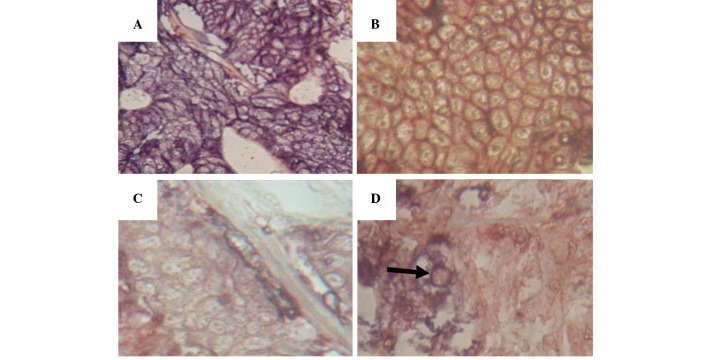
Immunohistochemical analyses of (A) EpCAM^high^/CD44^−^ cells, (B) EpCAM^low^/CD44^+^ cells, (C) EpCAM^low^/CD44^−^ cells and (D) EpCAM^high^/CD44^+^ cells in colorectal cancer. Magnification, ×400. EpCAM, epithelial cell adhesion molecule; CD44, cluster of differentiation 44.

EpCAM^high^/CD44^−^ cells were positive for BCIP/NBT staining but negative for AEC staining; EpCAM^low^/CD44^+^ cells were negative for BCIP/NBT staining but positive for AEC staining; EpCAM^low^/CD44^−^ cells were negative for the two stains; and EpCAM^high^/CD44^+^ cells were positive for the two stains (Fig. 1). The results were assessed according to Lin *et al* (9). Briefly, CD44 staining was detected mainly in the membrane and EpCAM staining was detected mainly in the cytoplasm. The results of EpCAM/CD44 cells proportion were classified into four groups, EpCAM^high^/CD44^+^, EpCAM^high^/CD44^−^, EpCAM^low^/CD44^+^, EpCAM^low^/CD44-. The proportion of EpCAM^high^/CD44^+^ tumor cells were defined as the percentage of cells positive for both blue-black and red staining, EpCAM^high^/CD44^−^ were positve for blue-black staining [5-bromo-4chloro-3-indolyl phosphate, p-toluidine salt (BCIP)/nitro blue tetrazolium (NBT); MaiXin Bio], EpCAM^low^/CD44^+^ were positive for the red staining [3-amino 9-ethylcarbazole (AEC); MaiXin Bio] and EpCAM^low^/CD44^−^ were negative for the two stains. The results of EpCAM^high^/CD44^+^ cells were based on the median value of their proportion checked for 10 visions under the microscope (BX51, Olympus Corporation, Tokyo, Japan). The labeling index (the percentage of positively stained cells) of each specimen was recorded and the values were averaged.

### Statistical analysis

SPSS 17.0 (SPSS Inc., Chicago, IL, USA) was used for statistical analysis. Differences in the means of continuous variables between the groups were analyzed using analysis of variance or t-tests. P-values from two-tailed tests are reported and in all analyses P<0.05 was considered to indicate a statistically significant difference.

## Results

### Expression of EpCAM/CD44 in samples of colorectal cancer and their corresponding liver metastases

**Figure 2:**
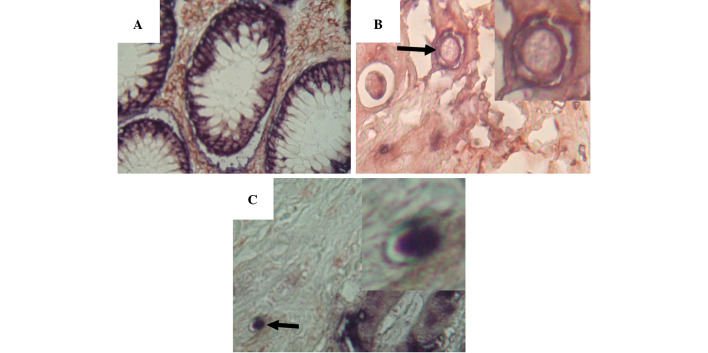
Double immunohistochemistry for EpCAM^high^/CD44^+^ cells showing (A) the absence of EpCAM^high^/CD44^+^ cells in normal intestinal mucosa, (B) EpCAM^high^/CD44^+^ cells in colorectal cancer and (C) EpCAM^high^/CD44^+^ cells in liver metastases. Magnification, ×400. EpCAM, epithelial cell adhesion molecule; CD44, cluster of differentiation 44.

Double immunohistochemical staining was used to detect the expression of EpCAM/CD44. The expression of EpCAM was predominantly cytoplasmic, whereas the expression of CD44 was primarily in the cytomembrane. EpCAM^high^/CD44^+^ double-positive cells were not present in the normal intestinal mucosa adjacent to colorectal tumors (Fig. 2A). However, in samples of colorectal cancer and their corresponding liver metastases, EpCAM^high^/CD44^+^ cells were visible (Fig. 2B and 2C). Double-positive cells accounted for 0.8–3.1% of cells in colorectal tumors. The expression of EpCAM/CD44 was significantly correlated with degree of differentiation, tumor stage, depth of invasion (Dukes’ stage) and metastatic status (P<0.05), while there was no correlation with gender, age or the magnitude of the tumor (P>0.05) (Table I).

## Discussion

Cancer stem cells have the capacity for infinite proliferation and self-renewal, and account for 0.3–2.2% of colorectal cancer cells. They are considered the cause of recurrence, metastasis and drug resistance in tumors (8). Therefore the identification of cancer stem cells and treatments targeting these cells facilitates elimination of the tumor.

EpCAM, a 40 kDa glycoprotein, functions as an epithelial cell adhesion molecule (11); its expression has been reported as localized to the epithelium along the basolateral surface of the majority of gastrointestinal tract mucosa. EpCAM can inhibit differentiation and promote proliferation (12). It is expressed in 85% of colorectal carcinomas and is one of the earliest tumor markers to appear that is involved in signal transduction, regeneration of tissue and other biological functions. EpCAM can upregulate the expression of the oncogene c-myc, induce acceleration of the cell cycle and promote proliferation (12). The overexpression of EpCAM enhances the proliferative and invasive capacities of tumors, while downregulation by RNA interference inhibits these functions (13).

CD44 is a multifunctional class I transmembrane glycoprotein located on the cytomembrane (14,15). As a cell adhesion molecule, CD44 is mainly involved in cell-cell and cell-matrix interactions (16). CD44 plays a vital role in the regulation of cell adhesion, growth, differentiation, migration and angiogenesis, and contributes to tumor progression by promoting invasion and metastasis (14,15). Knockdown of CD44 in primary colon cancer cell lines reduces clonogenicity *in vitro* and tumorigenicity *in vivo* (17). Schulenburg *et al* isolated CD44^+^ and CD44^−^ cells from colon cancer samples. The authors found that CD44^+^ cells had characteristics of stem cells and showed much higher capacity for proliferation and invasion than CD44^−^ cells (18). Thus, CD44 is considered a marker of cancer stem cells. In combination with other surface markers, CD44 can also be used to discriminate between a variety of cancer subsets (19).

Previous studies have revealed that the composite structure of EpCAM and CD44 can promote invasion and metastasis of tumors more strongly than any single adhesion molecule (20–22). In 2008, Marhaba *et al* proposed that EpCAM^high^/CD44^+^ cells are a marker of colorectal cancer stem cells (23), and Dalerba *et al* found that the EpCAM^high^/CD44^+^ phenotype of colorectal cancer cells has stem cell-like properties (4). The authors reported that 200–500 EpCAM^high^/CD44^+^ cells promoted tumorigenesis in non-obsese diabetic/severe combined immunodeficient mice, while no tumor was produced by injection of 10^4^ EpCAM^low^/CD44^−^ cells. Since the EpCAM^high^/CD44^+^ phenotype of colorectal cancer cells, which has stem cell-like properties, was confirmed (24), it has been regarded as an effective marker of colorectal cancer stem cells (25). The identification of cancer stem cells improves the understanding of tumorigenesis.

In the present study, 80 cases of colorectal cancer and their corresponding liver metastases were examined. EpCAM^high^/CD44^+^ cells were not present in the adjacent normal intestinal mucosa. However, in colorectal cancer tissue and their corresponding liver metastases, EpCAM^high^/CD44^+^ cells were visible. The percentage of double-positive cells was 0.8–3.1% in colorectal cancer, which is consistent with a previous study (9). Further analysis found that the percentage of EpCAM^high^/CD44^+^ cells in poorly differentiated tumors was higher than that in highly or moderately differentiated tumors. In addition, the percentage of EpCAM^high^/CD44^+^ cells in the T-stage 4 and Dukes’ D groups or in cases of metastasis was higher than that at other stages or in the group without metastases. Statistical analysis revealed that EpCAM^high^/CD44^+^ correlated with degree of differentiation, clinical stage, depth of invasion and metastasis. These results demonstrate that EpCAM^high^/CD44^+^ expression is significantly correlated with invasion and metastasis, and confirm EpCAM^high^/CD44^+^ cells as effective markers for colorectal cancer stem cells. These findings support the proposal that cancer stem cells may be the cause of recurrence and metastasis.

Based on the theory that cancer stem cells are the source of tumorigenesis, recurrence and metastasis in tumors, targeting cancer stem cells should effect the elimination of tumors. Specific drugs (catumaxomab and edrecolomab) targeting EpCAM have been developed, which can significantly reduce the size of tumor used alone or in combination with standard treatment, demonstrating the potential of such targeting strategies (26,27). Further study and the development of targeted drugs is required to elucidate the mechanisms of colorectal cancer stem cells and improve the clinical prognosis of colorectal cancer.

EpCAM^high^/CD44^+^, which is regarded as a marker of colorectal cancer stem cells, is significantly correlated with the invasion and metastasis of colorectal cancer, suggesting that this molecular marker may promote the progression of tumors.

## Figures and Tables

**Table I:** Expression of EpCAM/CD44 in colorectal cancer.

Variable	Samples, n	EpCAM^high^/CD44^+^ cells, %	P-value
Gender			1.000
Male	38	0.99±1.11	
Female	42	0.99±0.90	
Patient age, years			0.212
>60	44	0.84±1.04	
≤60	36	1.12±0.96	
Tumor magnitude, cm			0.051
≤5×5	50	0.80±0.73	
>5×5	30	1.31±1.29	
Differentiation			<0.001^a^
High	10	0.53±0.93	
Moderate	51	0.74±0.68	
Low	19	1.90±1.23	
Tumor stage			0.002^a^
T1+T2	18	0.49±0.43	
T3	28	0.80±0.43	
T4	34	1.41±1.00	
Dukes’ stage			<0.001^a^
A	16	0.42±0.39	
B	16	0.62±0.74	
C	22	0.89±0.66	
D	26	1.66±1.26	
Metastasis			<0.001^a^
Negative	32	0.52±0.59	
Positive	48	1.31±1.09	

a P<0.05.

EpCAM, epithelial cell adhesion molecule; CD, cluster of differentiation.
